# Role of Hippo Pathway Effector Tafazzin Protein in Maintaining Stemness of Umbilical Cord-Derived Mesenchymal Stem Cells (UC-MSC)

**Published:** 2018-04-01

**Authors:** Madhumala Gopinath, Rosa Di Liddo, Francesco Marotta, Ramachandran Murugesan, Antara Banerjee, Sushmitha Sriramulu, Ganesan Jothimani, Vimala Devi Subramaniam, Srinivasan Narasimhan, Swarna Priya K, Xiao-Feng Sun, Surajit Pathak

**Affiliations:** 1 Department of Allied Health Sciences, Chettinad Hospital & Research Institute (CHRI), Chettinad Academy of Research and Education (CARE), Kelambakkam, Chennai-603103, India; 2 Department of Pharmacology and Pharmaceutical Sciences, University of Padova, Padova, Italy; 3 ReGenera R&D International for Aging Intervention, Milano-Beijing, Italy-China, VCC Preventive Medical Promotion Foundation, Beijing, China; 4 Department of Gynecology and Pediatrics, Chettinad Hospital & Research Institute (CHRI), Chettinad Academy of Research and Education (CARE), Kelambakkam, Chennai-603103, India; 5 Department of Oncology and Department of Clinical and Experimental Medicine, University of Linköping, Linköping, Sweden

**Keywords:** Tafazzin, Stemness, Extracellular matrix, Proteoglycan, Oncoprotein

## Abstract

Tafazzin (TAZ) protein has been upregulated in various types of human cancers, although the basis for elevation is uncertain, it has been made definite that the effect of mutation in the hippo pathway, particularly when it is switched off, considerably activates tafazzin transcriptionally and thus this results in tissue or tumor overgrowth. Recent perceptions into the activity of tafazzin, have ascribed to it, a role as stem cell factor in mouse mesenchymal and as well as in neural stem cells. Being a downstream molecule in Hippo signalling, phosphorylation or dephosphorylation of tafazzin gene regulates its transcriptional activity and the stemness of mesenchymal stem cells. Commonly, extracellular matrix controls the stem cell fate commitment and perhaps tafazzin controls stemness through altering the extra cellular matrix. Extracellular matrix is generally made up of prime proteoglycans and the fate stabilization of the resulting lineages is surveilled by engineering these glycans. Tafazzin degradation and addition of proteoglycans affect physical attributes of the extracellular matrix that drives cell differentiation into various lineages. Thus, tafazzin along with major glycans present in the extracellular matrix is involved in imparting stemness. However, there are incoherent molecular events, wherein both tafazzin and the extracellular matrix components, together either activate or inhibit differentiation of stem cells. This review discusses about the role of tafazzin oncoprotein as a stemness factor.

## Introduction

 The cancer stem cells (CSC) generally acquire the features that are related to the normal stem cells. In the study of CSCs, the role of Tafazzin (TAZ) gene has been looked into in the past decade. TAZ serves to be a downstream effector in the Hippo signalling pathway and increased TAZ protein levels have been linked with several other human cancers including breast, thyroid and non-small lung cancer ^1^^-^^2^. Organ homeostasis and tissue regulation, through cell proliferation and apoptosis is controlled by a characteristic regulatory pathway, known as Hippo pathway, which was initially identified in Drosophila model ^3^. TAZ has been obtaining importance very recently as studies have shown that it is overexpressed in various cancers ^4^.

Research publications normally use TAZ symbol for WWTR1. Prospective CSCs show increased activity of TAZ through increased WWTR1 protein levels ^5^. The increase in the perception of cell biology, with respect to stem cells, has now led to the isolation of various tissue specific stem cells and identification of stemness properties in cancer cells. This paves way in the identification of several biomarkers that have the ability to convert cancer cells into CSC ^6^. CSCs can be described as the cells that are developed in tumors or hematological cancers which are homogeneous to usual stem cells, particularly with the ability to self-renew and give rise to most of the cell types found in certain cancer. However, the latent property of these CSCs regarding cellular and molecular mechanism and development of cancer is still poorly understood. TAZ generally plays a main role in clonogenicity (ability to form clones), non-adherent growth in vitro and tumor formation in vivo ^7^. The outrageous level of RNA expression of TAZ correlates with the shorter survival among colon cancer patients. Thus, it is only natural, that TAZ is strongly expressed in endothelium rich organs ^8^ as the mutation of Hippo pathway, in any one of the endothelial organs, can lead to cancer.

According to the unpublished data deduced by Pathak S et al., experiments underlay the fact that the TAZ protein expression is very significant when it comes to tissue formation as it was seen to be overexpressed in primary cancer cells. Here, we studied TAZ expression in two primary cancer cell lines (KM12C and SW480) and two metastatic cancer cell lines (KM12L4a and SW620). The TAZ expression was significantly higher in primary cancer cell lines compared to the metastatic cell lines. One possible explanation as recently shown by ^9^ Calon A et al is that TGF-β may active the tumor microenvironment to assist the colorectal cancer cells during metastasis. Varelas et al. ^10^ reported that TAZ and TGF-β signalling play vital role in morphogenesis and tumorigenesis by activating the Smad proteins as their downstream effectors. Therefore, possible over-expression of TGF- β exerts profound effect in down-regulating TAZ expression in metastasis.

**Regulation of Hippo signaling pathway**

TAZ/WWTR1 is a transcriptional co-activator acting as a downstream regulatory target in the Hippo pathway, which is a prominently conserved developmental pathway that has a major role in controlling organ size, suppression of tumor, regeneration of tissues and self-renewal capacity of stem cells. Besides, this pathway is formidably regulated by wide array of extracellular hormones which includes epinephrine, glucagon, sphingosine-1-phosphate, thrombin, etc. In mammals, the Hippo pathway comprises of serine/threonine kinases MST1/2 (mammalian Ste2-like kinases, Hpo orthologs) and LATS1/2 (large tumor suppressor kinase 1/2, Wts orthologs) ^11^. Current studies show that the inactivation of YAP after the detachment of cell instigates anoikis, which is a certain type of apoptosis that is suppressed in the cancer cells to assist cell survival and metastasis ^12^. The inhibition of YAP by cell contact signals is mediated by tight and adherens junctions ^13^. The YAP regulation mechanism is possibly significant for the role in cell contact inhibition. The Hippo pathway has recently appeared as a vital modulator of pluripotency in in vitro conditions. 

**Figure 1:**
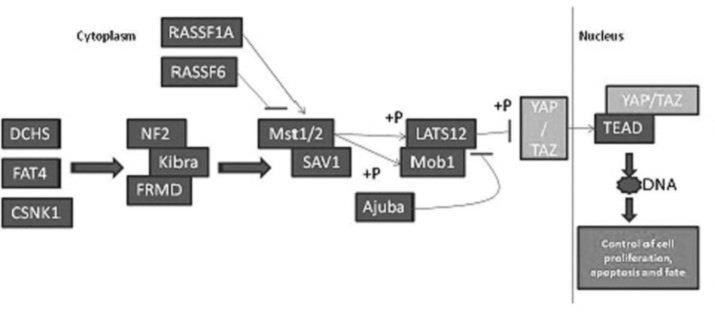
Hippo signaling pathway in Mammals. Source: Kegg software.

This pathway plays a significant role in controlling organ size (physiological) and tumorigenesis (pathological) sharing common cellular signaling mechanisms. Mst1/2 kinase complexes with Sav1 that phosphorylates and then activates Lats1/2 kinase which in turn initializes Mob1. This again in turn phosphorylates and inactivates TAZ/YAP, (Figure-1) ^14^^-^^15^. 

**Figure 2:**
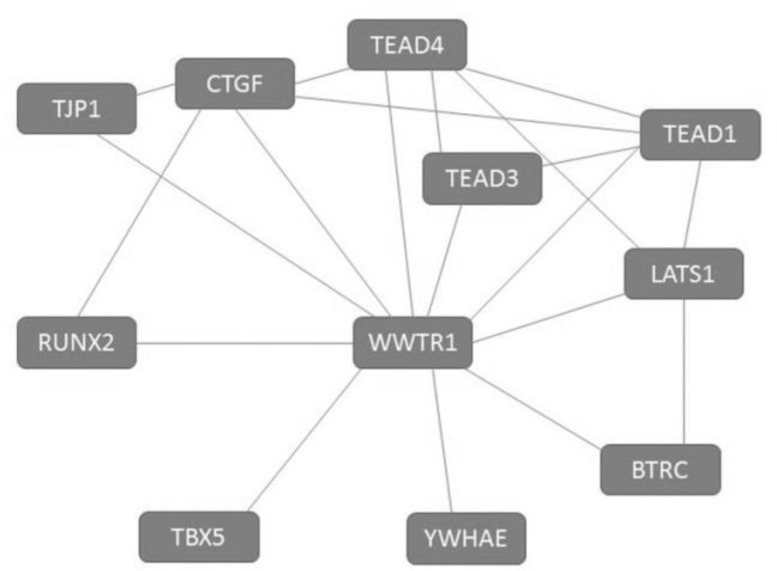
WWTR1 regulation. Source: STRING database

Triggering of the Hippo pathway results in the inactivation of TAZ and is segregated in the cytoplasm by binding to 14-3-3 and finally degraded in an ubiquitin-proteasome-dependent system. 9-fluorenone, a patented Hippo-YAP/TAZ inhibitor ^16^, inactivates YAP and TAZ which gets translocated to the cytoplasm. Cadherin receptors are down-regulated when TAZ is suppressed, leading to YAP/TAZ binding to the adherens junction and cell contact is immediately inhibited escorting to proteasomal digestion ^17^. Conversely, dephosphorylated YAP or TAZ act through TEAD family transcription factors or other transcription factors like Runx1/2, Smad, p73, Pax-3, ErbB4 and T-box transcription factor 5 (TBX5) to generate cell proliferation and organ growth^18^ (Figure-2). 

**Figure 3:**
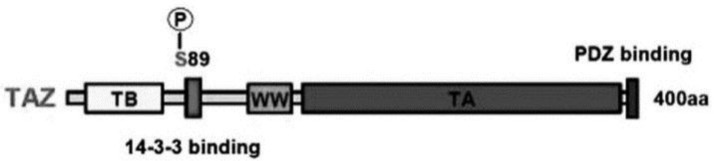
Structure of WWTR1/TAZ

The role of TAZ/WWTR1 in MSCs has become an imminent area in molecular biology and gaining its footprint that would eventually be a researcher’s riff. Chiefly, TAZ is a transcriptional co-activator of the Hippo pathway that depends on the subcellular translocation for their functioning ^19^. WWTR1 is a WW domain-containing transcriptional co-activator, which was first found as a 14-3-3 binding protein (Figure-3). TAZ play a major part in the regulation of various tissue specific stem cells and in repair of tissues. TAZ poses as a useful target in regenerative medicine ^20^ and its mutations are associated with diseases ^21^. Current findings incriminate the significance of Hippo pathway in homeostasis and also in development of epidermis.

**Mesenchymal Stem Cells derived from Umbilical cord (UC-MSC)**

Stem cells have the potential to self-renew, differentiate and engage in the repair and growth of damaged tissues ^22^. Even though MSCs have cell therapeutic values, their collection and yield pose great challenges to the physicians. The alternative sources include adipose tissue, umbilical cord (UC), umbilical cord blood (UCB) and embryo-derived tissues, etc ^23^^-^^31^. UC Wharton’s jelly (WJ) is a wholesome mix displaying high efficiency to convert the collection of cells to directly form MSCs, thus clinicians may be at an advantage by using WJ to refine their results in getting an efficient form of MSCs with maximum benefits pertaining to properties unique on its own ^32^. It is reported that WJ-MSCs appear as an idealistic approach to tap into stem cell resourcing as they have increased expression of undifferentiated human embryonic stem cell (ESC) markers when compared to BM-MSCs. They can also be instigated to be neural progenitors with higher efficiency compared to BM-MSCs and adipose-derived MSCs. (AD-MSCs) ^33^^-^^34^. MSCs themselves are susceptible to the effects of aging and diseases. Also if autologous MSCs are utilized, they may agonize from decreased potential to propagate, differentiate, home, engraft and exert immunosuppressive effects ^35^^-^^37^. MSCs were isolated initially from BM, while BM aspiration is a highly invasive procedure; it is detrimental for patients ^38^. Adipose tissue is thought to be a better alternative source than BM because it can be obtained by a less invasive procedure ^39^.

Of these adult stem cell sources, BMSCs and ADSC undergo fewer cell divisions before they reach senescence ^40^. However, UC and UCB can be a potent MSC as they lack this limitation, and moreover they are technically considered as biological wastes ^41^. Each year, 19.89 million births occur in India and 131.4 million occur worldwide ^42^, giving rise to an abundant source of MSCs. Moreover, the collection of UCB and UC is non-invasive, facing almost no ethical problems and above all they yield nascent stem cells. 

Researchers have found that UC cells acquired from umbilical vein’s sub-endothelial layer possibly differentiate in vitro into adipocytes and osteocytes ^43^^-^^44^, affirming the existence of plasticity in the population of fetal-derived tissues. But in ESCs, TAZ generally stimulates stemness directly as well as indirectly by imparting LIF signaling through the activation of the expressed genes that are truly responsible for the maintenance of pluripotent state both in in vitro and in vivo conditions.

 Time-honored principles already established in stem cell research include the fact that MSCs have a wide range of possibilities in differentiating into various lineages that enhance an important factor such as differentiation potential ^45^. It is important to initiate a dependable method of isolation, culture and characterization of human umbilical cord-derived MSCs (hUCMSCs) and to study its differentiation capacity ^46^. Serial passaging is the main approach to expand/differentiate stem cells in order to study the biological characteristics of hUCMSCs (WJMSCs). Basically, cell passages are split into three phases. They are early phase (passage 1-10), middle phase (passage 11-20) and late phase (more than 20). There is a need to evaluate MSC markers as recent studies have reported that certain senescence-related markers were seen to be up- regulated in the later phase of cell passaging ^47^. Most of the studies indicate that the MSCs derived from UCB appear to perpetuate their ancient characteristics ^48^.

**Understanding stemness**

Stem cells are considered in terms of the functional capacities which can only be evaluated by assessing the abilities of the cell, which may possibly alter its own features during assay procedures-a condition similar to the uncertainty principle in physics, which in summary tends to often utilize, differential equations to define stem cell dynamics: dS/dT=2Psm aS/T-aS/T=(2Psm-1) aS/T where Psm, a and T are considered to be independent of each other, hence can be subjected to various regulatory mechanisms. A certain fraction ‘a’ is actively propagating in the cell cycle time of T. Hence, per time unit aS/T cells enters mitosis. Of these cells, the fraction Psm self sustains. 

To provide a uniform genetic makeup, initial cells in culture form a niche that is reminiscent of a crypt, a code that the cells adhere to follow in order to communicate and live in an orderly fashion, so as to survive within its microenvironment. The crypt is known to comprise of six cell commanders that direct the cells to follow the cell’s disciplines that includes undergoing spontaneous apoptosis, release of growth factors, adherence, etc ^49^. 

Differentiation is said to be a qualitative modification in the cell phenotype, which is the result of onset of combination of new gene products, i.e., the noncyclic (new) changes in gene expression that leads to the functional competence ^50^. The ability to define a cell as differentiated clearly pivots on the sensitivity of the detection protocols. It may be identified by a change in the cell morphology or by the appearance of modifications in enzyme activity or composition of protein. Differentiation is commonly recognized by the detection of a novel protein, in this case, TAZ. 

**Functions involved in the regulation of TAZ**

TAZ is considered as the major output of the Hippo signaling pathway and appears at the middle piece of a signaling nectere, by which cells control their behavior according to their shape, spatial location and growth factors ^51^. TAZ is present both in the cytoplasm and in the nucleus, where they modulate the transcription of gene. The ECM that makes up for the extracellular environment acts as a plinth that helps in the interaction between the biological and physical components which possess the capacity to blend in zeitgeber signals influencing cellular phenotypes ^52^. Scribble is a cell-polarity determinant that combines with TAZ and is found predominantly in epithelial cells, loss of which leads to the disruption of the ability to inhibit TAZ. This instigates EMT that is directly proportional to the stem cell acquisition. It has been studied that YAP, an isomer of TAZ, becomes transcriptionally active and seems to be expressed in large proportions in stem cells where it is active in the nucleus ^53^. The effect of TAZ subcellular localization is predominant in the physical properties of the ECM ^54^.

**(a)** **EMT and post translational modifications**

EMT is a complex transdifferentiation program that is instrumental for the acquisition of stemness ^55^^-^^56^. Overexpression of TAZ can instigate anchorage independent growth, EMT of immortalized mammary and also pancreatic epithelial cells in vitro ^57^. TAZ regulation of ECM may be associated with the regulation through VE- and N-Cadherin. N-cadherin adhesive interactions modulate matrix mechanosensors and determine the fate of mesenchymal stem cells ^58^. The biomarkers of EMT, vimentin and N-cadherin were down-regulated when TAZ was suppressed ^59^.

Post-translational modifications of TAZ (PTMs) include activation and deactivation, subcellular localization of signaling proteins which facilitates the initiation, amplification and also transduction of signals. PTMs include ubiquitination, sumoylation, acetylation and protein methylation ^60^. 

We hypothesize that as and when a stem cell loses TAZ molecule in the cytoplasm due to post- translational modifications, the cell loses its stemness. Even though the TAZ effector molecule can be dephosphorylated and brought back to the nucleus ^61^, the stemness is not reverted as the cell starts to differentiate.

**(b)**       **Cell Mechanics and TAZ subcellular shuttling**

Cell mechanics and the status of the cytoskeleton are accurate overarching signals that cells utilize to make necessary decisions such as multiplication, differentiation and maintenance of stem cells ^62^^-^^65^. Activity of TAZ inhibits differentiation, but we need mechanotransduction proofs to substantiate this property ^66^^-^^67^. Strikingly, these mechanical and cytoskeletal inputs represent a central mechanism to control TAZ activity. A simple classic binary cellular decision-proliferation versus differentiation is well-known for being intensely influenced by cell shape ^68^^-^^69^. When a single cell is allowed to stretch over the ECM, the cytoskeletal adaptation to spread the cell shape (including formation of F-actin stress fibers) causes TAZ nuclear accumulation and activation that promote cell proliferation and inhibit differentiation ^70^. When ECM adhesive region is brought down to a smaller area, the morphology can be altered to yield a smooth edge that pushes the gene away from the nucleus and initiates differentiation ^71^^-^^72^.

**Figure 4:**
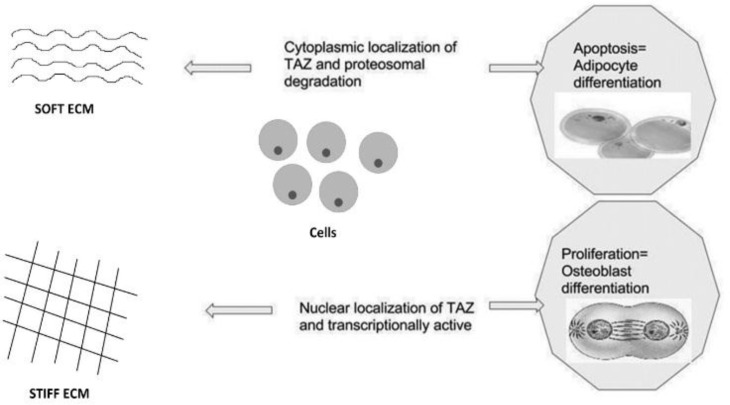
Subcellular localizations of TAZ and its effect

Subcellular translocation provides new capabilities for TAZ to manifest itself into a stem cell marker during transcriptionally active phase. The ability of YAP and TAZ to control organ growth and size is obvious, but, at the same time, the intricate mechanism involved for this behavior is not completely understood ^73^. Cells grown on compliant substrates or space limiting substrates exhibit cytoplasmic localization of TAZ correlating with the results from Varelas X et al., 2014 (Figure-4) where a stiff ECM-stretched matrix increases the nuclear localization and a small adhesive area leads to TAZ cytoplasmic localization. In the nucleus, the TAZ/YAP-SMAD2/3 complex binds to TEAD transcription factors, as well as to the core stem cell regulator OCT4 and jointly arbitrates the pluripotent state ^74^. Mechanistically, this complex convenes with factors that make up the nucleosome remodeling and deacetylation (NuRD) complex to buffer the expression of pluripotency genes and suppress genes that describe mesendoderm. The crux of the correlation here is whether TAZ localization affects the ECM or ECM affects TAZ localization or if both are complementary reactions as the consequence affects stemness ^75^^-^^76^.

**Genes involved in the regulation of ECM: A distinguished milieu of TAZ & Proteoglycans**

Heparan sulfate (HS) is a proteoglycan (HSPG) and a linear polysaccharide found in the extracellular environment in most of the animal tissues and makes up for the cellular niche where along with HS chains various cell signalling molecules, growth factors, cytokines and other ECM components are attached to the ECM proteins. This in turn makes up the matrix biology and eventually takes part in the molecular events. It also acts as a morphogen gradient displaying co-receptor functions and takes part in the cascade pathways. Developmental processes, angiogenesis, blood coagulation and cancer migration are regulated when HS binds to various protein ligands ^77^^-^^78^. Glycan-Glycan-Cell interaction has been found to have a predictable effect on these proteoglycans on the potent cell surface proteins, compromised in regulating the extracellular diffusion and efficiency of signals ^79^. 

The similarity in functionality paves way for new panels leading to the intracellular complementary regulatory networks that are increasing the availabilities of growth factors to be presented in the milieu to achieve the stemness-goal. These genes along with ECMs proteoglycans has been extensively studied in relation to the Hippo core kinase complex and its role in organ size control, stem cell fate and cancer ^80^^-^^82^. The following factors serve as a clue to the above study. The genes discussed here are involved in the TAZ regulation, are also co-dependent and expressed in ECM cascade signalling processes which are interdependent on the proteoglycans present in the ECM. 

**(a)** **FGF and VEGF**

In cell culture, to attain a confluent mass of cells, the proliferation media is generally supplemented with growth factors like FGF and VEGFs ^83^^-^^84^. 

HS regulates the number and asymmetric division of germline stem cells (GSCs) in the Drosophila testis which also resulted in the knowledge about the functionality involved in Drosophila and vertebrate that regulates Wnt, bone morphogenetic proteins (BMPs) and FGF signals ^85^^-^^86^.

As noted by Varelas et al, the TAZ coding gene entertains the relationships with morphogenetic signals such as Wnt and growth factors. They are also regulated by Rho, GPCRs and mevalonate metabolism.

**(b)** **TGFβ superfamily**

As members of the TGFβ superfamily, BMPs play an important role in stem cell proliferation, skeletal and extra skeletal development and differentiation during development ^87^^-^^92^. TAZ binds with different transcription factors like the TEA- DNA-binding domain (TEAD) transcription enhancing factors and acts as a mechano-regulator of TGF SMAD signalling in renal fibrogenesis ^93^^-^^96^.

It has been studied in in vitro conditions that HS drives chondrogenesis through inducing TGF mechanotransduction ^97^. HSPGs, those expressed in the niche (cap cells), activate BMP signalling in-trans by directly contacting GSCs. This trans-core receptor activity of HSPGs can explain the contact dependence of GSC maintenance ^98^.

**(c)** **Runx2 and PPAR gamma**

Runx2 and PPAR gamma are the essential transcription factors that drive MSCs to differentiate into either osteoblasts or adipocytes respectively. TAZ co-activates Runx2-dependent gene transcription while repressing PPAR gamma-dependent gene transcription ^99^. In order to view this, scientists worked on attuning its expression in cell lines in vivo and in turn observed most significant changes in its potential. Cerebellar granule cell precursors express members of the Runx family and that the expression of those genes can also be controlled by glial factors. Studies show that the expression of all genes studied is modified during the postnatal development and treatment of precursors with glial factors demonstrates that the expression of heparan sulfate proteoglycan genes as well as genes encoding heparan sulfate modifying enzymes can be regulated by the microenvironment, reflecting the intricate relations between neuron and glia ^100^.

**(d)** **SOX2**

Expression analysis of the Hippo cascade demonstrates a role of SOX2+ in pituitary stem cell development. This stemness marker is necessary for MSC characterization ^101^. According to the studies on extracellular matrix gene regulation, ^102^ SOX transcription factors are positive regulators for the ECM genes. 

Studies on neural stem cells resulted in changes in distribution of cell in the cortical layer due to the increased stemness of infected cells; YAP-expressing cells were co-labeled with SOX2, a neural stem cell marker and YAP/TAZ thereby increased the frequency and size of neurospheres, indicating intensified self-renewal and proliferative ability of neural stem cells ^103^.

Pertaining to this, cell-surface HSPGs were provided as novel markers of human neural stem cell fate determination ^104^. 

**Critical interpretation of an oncogene as a stemness factor**

**Figure 5:**
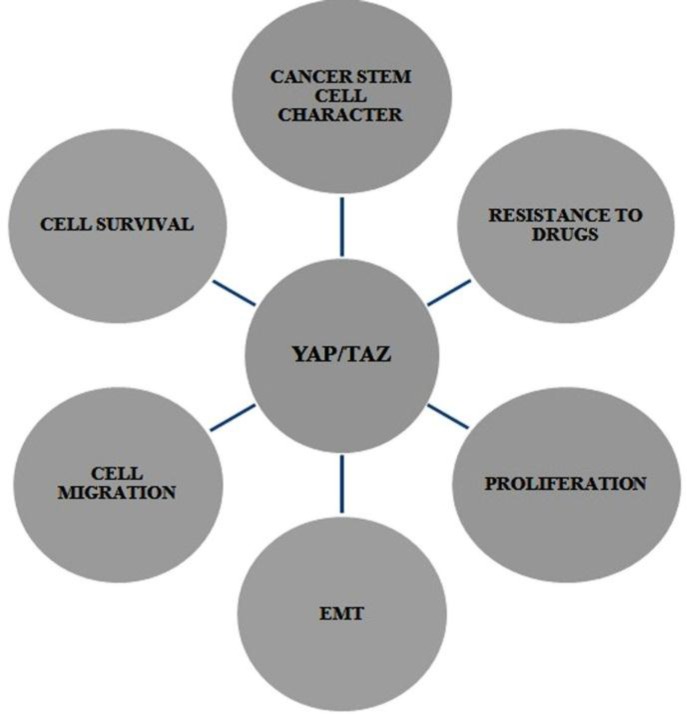
Cellular functions of TAZ in Cancer

**Figure 6:**
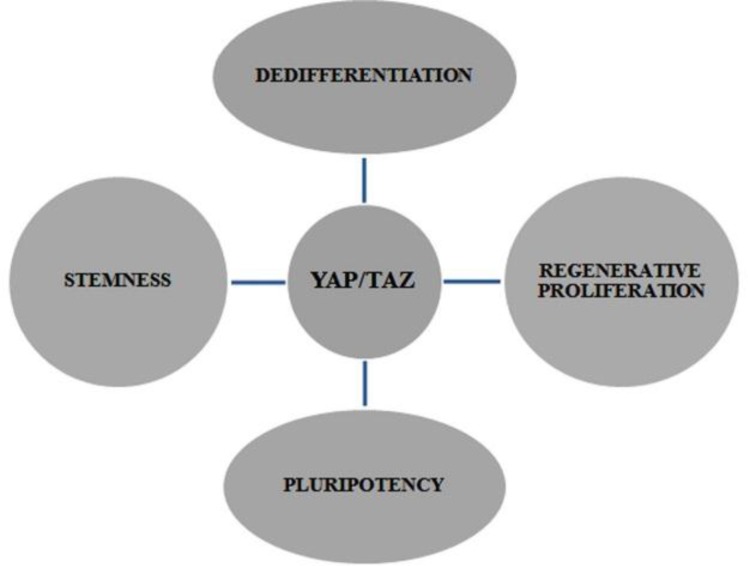
Cellular functions of TAZ in stem cells

As described above, TAZ protein has been recognized as an oncoprotein, but its primary action as a stemness regulator has only lately begun to be studied. So, we discussed that the Hippo signaling functions as an organ regulator in normal physiological conditions, but when the mutation occurs it leads to cancer, thereby *TAZ* stop receiving signals and Hippo pathway remains switched off. This leads to uncontrolled growth as TAZ is transcriptionally active. In the same environment, the cell contact inhibition is deactivated; hence cell-cell contact does not assert itself in the mutated pathway and thus leads to increased tumor growth. (Figures 5 (a) and 6)

According to Ramos A et al., YAP has been implicated as a stemness gene. Previously, Lian J et al. and Tamm C et al. ^105^ have shown that the presence of YAP is required mainly for the maintenance of pluripotency in mouse embryonic stem (ES) cells and Knockdown of YAP in mouse ESCs leads to the loss of OCT4, SOX2 and consequent differentiation. Parallels between ES and cancer cells include shared similarities in transcriptome signatures, indefinite proliferation and an undifferentiated state ^106^. 

In 2006, Yamanaka et al. demonstrated the induction of pluripotent stem cells from mouse embryonic stem cells by introducing four factors such as Oct3/4, c-Myc, SOX2 and Klf4 under ES cell culture conditions ^107^. c-Myc is a known oncogene, which is up regulated in several tumors that has shown to contribute to the long-term maintenance of ES phenotypes ^108^. Yamanaka et al. have pointed out to the fact that the use of an oncogene such as c-Myc for clinical applications may not be suitable and the processing of which requires alterations and specialized environment. Nevertheless, the finding is extremely important.

TAZ is a YAP counterpart and plays a critical role in contributing to the phenotypes shared by ES and CSC, making Hippo-YAP/TAZ pathway an attractive target for stem cell study.

## CONCLUSION

 The pluripotency in stem cells has been supported by many molecular signalling processes, transcription factors, signal transduction pathways and epigenetic regulators which have attracted many arguments and ideas in stem cell research. There has been striking results that has advanced the science of stem cells to a greater level but lacks novel factors influencing this area. In this article, we reviewed that the TAZ genetic studies on gene-knockout mice indicates an eminent role as a fundamental supporter of organ growth and metastasis in epithelial tissues such as the liver, skin, ovaries and soft tissues. In human and mouse tissues, TAZ is active only in basal cells and is vital for basal cell maintenance during homeostasis condition. Accordingly, the loss of TAZ affects mammary gland development, leading to an imbalance of luminal and basal populations as well as branching defects. 

In cancer, it may be possible that once cell differentiation takes place, the cancer stem cell regulators are inactivated and do not function until molecular signalling is initiated. The recurrence of cancer takes place due to activation/inactivation of respective pathways, coordinating with oncogenes already existing in the body like the one that codes for TAZ protein which is up- regulated only when the pathway is mutated. This becomes very interesting as TAZ gene’s basic function is to confer a trait that provides stemness, but somehow mammalian cancer cells augment this epigenetic regulator and present a cancer phenotype. Nevertheless, TAZ is a molecular determinant or a signature of biological properties and functions linked with mesenchymal stem cells.

This review emphasizes the fact that the transcriptionally active TAZ seen in the nucleus might be a co-activator of stemness and is seen to be expressed in stem cells. TAZ moves on with its function to direct the stem cell fate in order to attain different lineages according to the ECM morphology. By adding factors such as Bovine serum albumin that enhances localization of TAZ gene in the nucleus, it might be possible to keep the stemness intact for a time period longer than the normal. The escape of TAZ into the cytoplasm means loss of TAZ which leads to proteasomal degradation and loss of stemness. 

While reviewing various signalling factors, there seemed to be an underlying link which has not been mapped, but TAZ and the proteoglycans present in the ECM are involved in it. So, it is quite likely that the main factors involved in stem cells, dependent on the physical properties of the ECM governed predominantly by TAZ gene, fate entirely. These results suggest that TAZ acts as a powerful co-activator in providing stemness. However, further analysis, experiments and critical studies are required to validate this claim. The existence of unidentified regulators and downstream transcription factors of TAZ is needed to be explored. The core signalling cascade from Hpo (MST1/2) to Yki (YAP) is well understood, but the upstream components, signals and regulatory mechanisms of the Hippo pathway have been partially determined (related to cell polarity, adhesion, mechanotransduction, as well as diffusible signals acting through GPCRs) and many key questions remain to be addressed. Furthermore, the function of YAP/TAZ has been scrutinized in only a few cell types including embryonic stem cells (ESCs) and induced pluripotent stem cells (iPSCs). Thus, additional studies are required to uncover the physiological roles of YAP/TAZ in a broad range of tissue-specific stem cells and various types of mesenchymal stem cells for clinical applications in the field of regenerative medicine. In our further studies we will pay more attention to Hippo pathway in regulating stem cell biology, tissue development and regeneration. 

## Abbreviations

CSC-Cancer Stem Cells, ECM-Extra Cellular Matrix, MSC-Mesenchymal Stem Cells, UCB-umbilical cord blood, UC-umbilical cord, BMSC-Bone marrow derived Mesenchymal Stem Cells, ADSC-Adipose-derived Stem Cells, hUCMSC-human Umbilical Cord-derived Mesenchymal Stem Cells, WJMSC-Whartons jelly-derived Mesenchymal Stem Cells, ESC-Embryonic Stem Cells, YAP-Yes Associated Protein, HSPG-Heparan sulfate Proteoglycan, EMT-Epithelial Mesenchymal Transition, PTMs-Post Translational Modifications, WWTR1- WW domain containing transcription regulator 1, MST1/2-mammalian Ste2-like kinases, LATS1/2-large tumor suppressor kinase ½, TEAD- TEA DNA-binding domain, TGF- Transforming Growth Factor, GSC- Germline stem cells, BMPs- bone morphogenetic proteins, SOX2- (Sex determining region) box-2.
